# Characterization of the daily and circadian valve behavior of the European flat oyster *Ostrea edulis*

**DOI:** 10.1038/s41598-025-98746-x

**Published:** 2025-04-29

**Authors:** Alexandre Le Moal, Damien Tran, Laura Payton, Bernadette Pogoda, Bettina Meyer

**Affiliations:** 1 Institute for Chemistry and Biology of the Marine Environment, Carl von Ossietzky University of Oldenburg, 26111 Oldenburg, Germany; 2 Section Polar Biological Oceanography, Alfred Wegener Institute Helmholtz Centre for Polar and Marine Research, Am Handelshafen 12, 27570 Bremerhaven, Germany; 3 Univ. Bordeaux, CNRS, Bordeaux INP, EPOC, UMR 5805, Arcachon, F-33120 France; 4 Shelf Seas Systems Ecology, Alfred Wegener Institute Helmholtz Centre for Polar and Marine Research, Bremerhaven/Helgoland, Germany; 5 Helmholtz Institute for Functional Marine Biodiversity at the University of Oldenburg (HIFMB), Im Technologiepark 5, 26129 Oldenburg, Germany; 6 GDR2202 - Lumière & environnement nocturne (LUMEN), UMR 5602 GÉODE, CNRS, University of Toulouse - Jean Jaurès, F-31058 Toulouse, France

**Keywords:** *Ostrea edulis*, Oysters, Daily rhythm, Circadian rhythm, Valve behavior, Free-running, Marine biology, Behavioural ecology, Environmental impact, Ecophysiology

## Abstract

Marine organisms exhibit a multitude of biological rhythms synchronized with the interactions of the sun-, earth-, and moon cycles. However, the biological rhythms in bivalves remain poorly studied. This study focuses on the native European flat oyster (*Ostrea edulis*), an endangered species of coastal ecosystems and a key organism in restoring of biogenic reef habitats. We aim to determine whether a molecular endogenous circadian rhythm exists in *O. edulis* and to characterize its daily expression. To address these questions, the oysters’ valve behavior, as an output of the circadian clock expression, was recorded under different light conditions and free-running regimes using non-invasive valvometry. This work demonstrates the existence of a circadian clock mechanism that generates a labile behavioral circadian oscillation under free-running conditions. In light: dark conditions, a diel rhythm appears nocturnal, synchronizable to a shift of light phase, and remains unmodified whether the oysters are fed or not. This rhythm anticipates light: dark changes, indicating its endogenous origin. Finally, when exposed to artificial light at night the daily behavior is disrupted. This study characterizes the circadian behavioral rhythm of *O. edulis*’s as plastic and labile. This plasticity would be advantageous in terms of ecological adaptability but increases sensitivity to anthropogenic pressures such as light pollution.

**Supplementary Information:**

The online version contains supplementary material available at 10.1038/s41598-025-98746-x.

## Introduction

The native European oyster *Ostrea edulis* (Linnaeus 1758) is a filter feeder formerly abundant in Europe from the North Sea, along the Atlantic coast and other European coastal waters including the Mediterranean and the Black Sea^1^. *O. edulis* is a biogenic reef builder that plays a key ecological role and provides many ecosystem services (e.g. substrate formation and biodiversity enhancement)^2^. Over the 20th century, stocks of *O. edulis* have been severely depleted by overfishing and additional anthropogenic stressors, such as invasive diseases and is now one of the most threatened marine habitats in Europe^3,4^. However, in recent years, conservation and active restoration of European oyster habitats across its former distribution range have become a major focus of ecological restoration efforts^5,6^ to take advantage of the wide-ranging ecosystem functions and services this species and it reef habitats provide^2^. Despite these recent efforts, knowledge of the general physiology of *O. edulis* has primarily centered on reproductive aspects. Notably, chronobiological studies of their behavioral and physiological traits are still lacking, which is crucial for understanding their ability to adapt to and fit within their cyclic and fluctuating environment.

Life on Earth involves exposure to cyclical environments that are related to the rotation of the Earth on its axis, the Earth’s trajectory around the Sun, and the Moon’s orbit around the Earth, as well as the interaction among these forces^7^. To maintain harmony with their environment, organisms have evolved biological rhythms that enable anticipation and synchronization with their biotope^8^. These biological rhythms are generated by an internal clock present in each cell of an organism^9^. It consists of a self-sustained auto-regulatory network of transcriptional and translational feedback loops. These ancestral and endogenous clockworks are entrained by cyclic and reliable environmental factors called *zeitgebers* (time givers), such as light that synchronize the circadian clock producing daily rhythm (~ 24 h)^9–11^. This clock mechanism facilitates the temporal organization of many metabolic, physiological and behavioral functions of organisms^12,13^. In the absence of *zeitgebers*, the clock is termed «free-running» and expresses its endogenous period, which is close but not identical to the environmental cycle^14,15^. By convention, rhythms with periods ranging from 20 h to 28 h are considered as circadian.

Studies on biological rhythms of marine organisms are increasing but they still lag behind those of their terrestrial congeners. Many of these behavioral rhythms are endogenous, controlled by molecular clock(s) and synchronized by cyclic environmental cues driven by solar and lunar light entrainments but also tides. In marine bivalves, studies of biological rhythms remain scarce. A good proxy for biological rhythms is the shell’s movement, the valve activity behavior^16,17^, which is closely related to respiration, nutrition, and reproduction. Previous in situ studies on bivalves have revealed that valve behavior could exhibit a multitude of rhythms such as tidal, daily, lunar and annual ones^18–22^. Regarding the characterization of the clock mechanisms that underlie these rhythms, few studies have been conducted under controlled conditions, particularly on the Pacific oyster *Crassostrea gigas*^17,23^ and the Blue mussel *Mytilus edulis*^24^. Recently, a preliminary study has investigated the daily valve activity of *O. edulis*^25^. However, the characteristics of its rhythm and the underlying mechanism have not yet been described.

Anticipation of environmental cycles allowed by a clock mechanism provides an advantage for animal fitness^26^. However, while endogenous clock systems improve species fitness under steady ecological conditions, a too robust clockwork could limit species’ adaptation potential in a changing world due to global warming^27^. Furthermore, anthropic pressures, such as the recent threat of artificial light at night (ALAN) are increasing in all coastal ecosystems^28^. By masking natural variations of light, ALAN can disrupt the marine organisms’ light perception and affect organisms’ biological rhythms and, thus, their physiology. This disruption can have cascading effects on critical physiological processes such as feeding and reproduction, potentially leading to population declines^29,30^. This is consequently, in the context of re-introduction this endangered species, it is essential to investigate the chronobiology of the native European oyster *O. edulis* in its environment.

To achieve this goal, we first characterized the daily rhythm of valve activity using the high frequency non-invasive (HFNI) valvometer biosensor^31,32^. We characterized this daily rhythm in terms of diurnal/nocturnal modality, robustness, synchronization by light/dark cycles, and response to food availability. Secondly, we investigated the existence of an underlying circadian clock mechanism responsible for this daily rhythm by studying valve behavior under free-running conditions and its anticipatory patterns under light/dark conditions. Thirdly, we tested whether ALAN at low intensity (5 lx) could disrupt the circadian rhythm of *O. edulis*.

## Materials and methods

### Experimental model and general conditions

All research detailed in this study complied with French law, and the experiment was conducted according to international ethical standards. The experiment took place in Arcachon, France (at the Marine Station, 44° 39′ 48″ N, 1° 9′ 49″ W) over a period of 97 days, from March to June 2023, involving 32 European native oysters *O. edulis* (74.7 ± 1 mm shell length and 72.5 ± 1 mm shell width, mean ± standard error (SE); approximately 8–10 years old). These European native oysters were collected at Loch Ryan in South West Scotland (54° 59′ 10″ N, 5° 03′ 18″ W) (Rossmore Oysters Ltd.) and acclimated for 4 months under natural light conditions in a continuous flow-through system using natural seawater from Arcachon Bay, supplement with a food supply of microalgae (*Chaetoceros calcitrans)*.

### Experimental setup

**Fig. 1:**
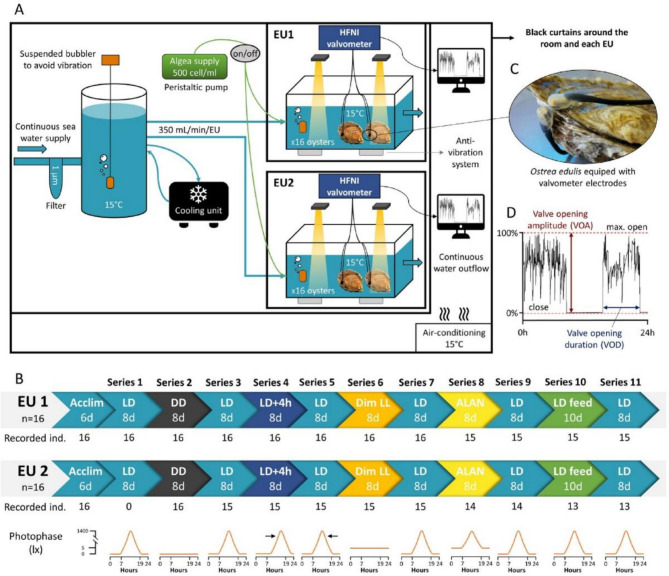
Experimental setup and protocol. (**A**) Experimental setup to investigate the circadian rhythm of *O. edulis*. (**B**) Experimental protocol. Timeline of the 11 series to which the oysters of both EUs are exposed successively. (**C**) Zoom of *O. edulis* equipped with HFNI valvometry electrodes for valve behavior recording. (**D**) Example of a daily individual valve activity showing the two behavioral parameters chosen in this experiment, i.e. the valve opening amplitude (VOA) and the valve opening duration (VOD). EU: experimental unit. HFNI: high frequency non-invasive.

Throughout the entire experiment, the oysters were isolated from external vibrations using an antivibration system and an isolated blind room to minimize any external influences on their valve behavior (Fig. 1A). Experiments were performed in two distinct experimental units (EU1 and EU2) (L × W × H:74.8 × 54.8 × 40.8 cm), containing approximately (~) 150 L of seawater, which was continuously supplied with filtered (< 1 μm) and oxygenated seawater at a flow rate of 350, maintaining a constant composition of 15 ± 0.1 °C, pH = 7.9 ± 0.1, salinity = 33.1 ± 0.1‰, mean ± SE. A 180 L retention tank was situated between the seawater supply and the two EUs to homogenize the seawater and prevent potential environmental cycle cues. The experimental setup and each EUs were surrounded by opaque black curtains to shield the experiment from external light contamination (Fig. 1A).

### Experimental protocol

After 6 days of acclimatization in experimental design, eleven series (lasting 8–10 days) were conducted based on various photoperiods and other experimental cues (Fig. 1B). All times are presented in local time, i.e. UTC + 1 (Coordinated Universal Time). The 11 series comprised: reference 12 L:12 D (8 d, photophase from 07:00 to 19:00 h, series 1, 3, 5, 7, 9, 11); D: D (8 d; series 2); 12 L:12 D with a 4 h-phase shift (8 d, photophase from 11:00 to 23:00 h; series 4); dim L: L (8 d; series 6), 12 L:12 ALAN (8 d; photophase from 07:00 to 19:00 h; series 8), and 12 L:12 D with food supply (10 d, photophase from 07:00 to 19:00 h; series 10). Throughout all series the oysters were in unfed conditions, except for the series 10, where the oysters were continuously supplied with a solution of microalgae *Chaetoceros calcitrans* at a flowrate of 100 mL/min/EU to maintain a concentration of ~ 500 cell/mL in each EU.

Illuminances were measured underwater at the oysters’ depth in lux (lx) in each EU using a handheld spectroradiometer (Blue-Wave UVN-100, StellarNet Inc.). Equivalent irradiance in µE/m²/s was also measured using a portable radiometer (MICRO Class, Profiling (in-water), E (irradiance) PAR sensor, Biospherical Instrument Inc.). During the photophase of all the 12 L:12 D series, the light intensity varied gradually to mimic the natural light cycle using programmable white (413–688 nm, peak at 551 nm) LED light bars (MH3SP3 DSunY). The maximum light intensity during daytime was 1406.1 ± 136.2 lx (mean ± SE) corresponding to 28.99 µE/m²/s in irradiance, occurring between 12:30 and 13:30 h, except for series 4, where the maximum of light intensity was between 16:30 and 17:30 h. During the scotophase, the light intensity was below the detection limit of the spectroradiometer (< 0.05 lx). During the dim L: L condition (series 6), the light intensity was maintained continuously at 5.67 ± 0.34 lx (0.15 µE/m²/s), corresponding to a dim light exposure, to minimize masking effects. During the ALAN (series 8) condition of 12 L :12 ALAN, the oysters were exposed to 5 lx during the scotophase (Fig. 1B).

### *Ostrea edulis* valve behavior measurement and representation

The valve activity of 16 oysters per EU was measured using high-frequency non-invasive (HFNI) valvometers. They consist of a pair of lightweight electrodes (< 100 mg) designed to minimize disturbance of the oyster behavior. The electrodes are glued on each half-shell and each electrode is linked to the HFNI valvometer by a flexible wire allowing undisturbed oyster valve movement (16 oyster monitored by valvometer) (Fig. 1C). An electromagnetic current is generated between the electrodes, allowing the measurement of each oyster’s valve activity every 4.8 s. Further details are available on (Tran et al., 2023)^32^ and (Le Moal et al., 2023)^22^. Raw daily recordings are processed using Labview 8.0 (National Instrument, Austin, TX, USA). It is important to note that for series 1, EU2 data are missing due to electrical failure (Fig. 1B).

To analyze the behavioral rhythm, two parameters of valve activity were measured: the valve opening duration (VOD) and the valve opening amplitude (VOA) (Fig. 1D). For each hour the individual VOD and VOA are measured. VOD is established from an opening threshold (5% of VOA) setting the state “open” or “closed” of the animal. If the oyster is continuously open for an hour, the percentage of hourly VOD is 100%. On contrary, if it does not open at all for an hour, the hourly VOD is 0%. Individual VOA of each oyster is reported each hour as a percentage where 100% indicates that the valves opened at their maximum amplitude during the whole hour and 0% indicates that the valves were closed during the whole hour, in between all the values of amplitudes are possible.

The mean hourly VOA and VOD are represented as double-plotted actograms and heatmaps, where each line represents two days (Fig. S1). In an actogram, hourly activity levels superior to the daily median are represented by dark blue (VOD) or red (VOA) bars, whereas hourly activity levels inferior to the daily median are represented by light grey bars. In heatmaps the mean hourly VOA and VOD are represented according to a color scale from 0 to 100%, divided into ten intermediate ranges.

### Chronobiological analyses

Chronobiological analyses were performed using the software Time Series Analysis Serial Cosinor 6.3. Several steps were necessary to validate a significant rhythm. First, to assess the data quality, the absence of random distribution in the data set is checked using an autocorrelation diagram, and the lack of a stationary phenomenon is evaluated through a partial autocorrelation function calculation^33^. Next, the data were examined for periodicity ranging from 5 to 35 h using spectral analyses and the Lomb and Scargle periodogram^34^. This periodogram provides a significant threshold (*p* = 0.95). Finally, rhythmicity is validated by the Cosinor model employing the period identified by the Lomb and Scargle periodogram^35^. For a specified period, the model is expressed as Y (t) = Acos (πt/τ + φ) + M + ε (t), where A represents amplitude, φ is the acrophase, τ is the period, M denotes the mesor and ε signifies the relative error. Two critical tests confirm the calculated model and the presence of a rhythm: the elliptic test must be rejected, and the probability for the null amplitude hypothesis must be < 0.05. A chronobiometric parameter was calculated: the percent rhythm (PR, %) and the proportion of cyclic behavior explained by the model. For each series, binary VOA and VOD datasets represented in actograms were examined for rhythmicity. These analyses were conducted at the group level (EU1 + EU2, *n* = 32 individuals) and the individual level. Only rhythms within the range of 20–28 h were identified in this study. To address multiple testing in chronobiological analyses, a Benjamin-Hochberg *p* < 0.05 was considered significant. Raw results of individual analyses are available in Supplementary Table 1.

## Results

### Actograms and chronobiological analyses of valve activity

**Fig. 2:**
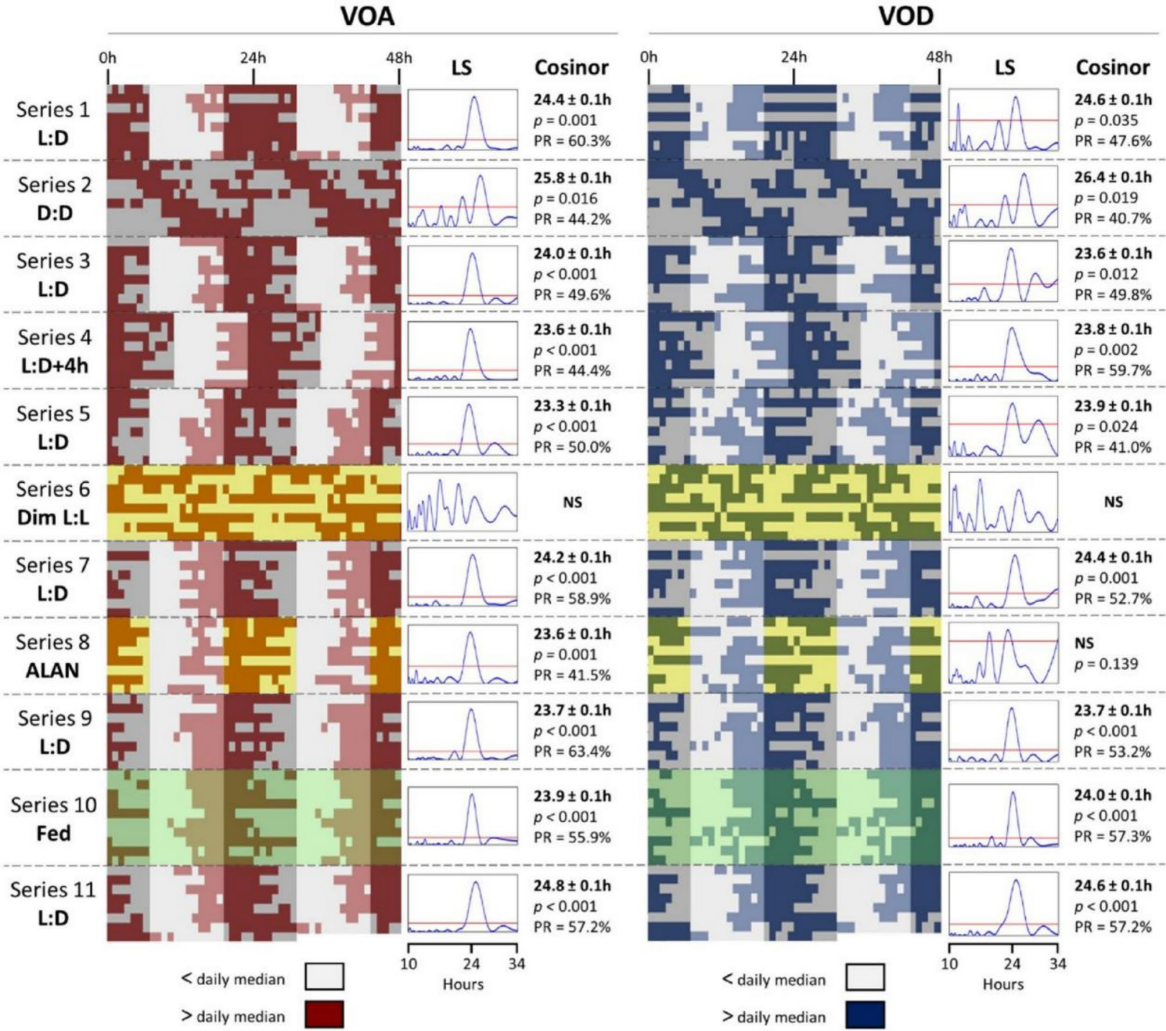
Actograms and chronobiological analyses of *O. edulis* valve activity. Actograms of mean VOA (left panel) and VOD (right panel) during the 11 series, 97 days in total (*n* = 16–32). Each actogram of each series is associated the Lomb & Scargle periodogram (LS) performed on binary data and characteristics of the Cosinor model: the period (h) ± SE, the *p-value* and the percent rhythm (PR, %). The red line on the LS periodogram indicates a threshold of significance (*p* = 0.95). The rhythm is considered significant for the Cosinor model with *p* < 0.05. On actograms, dark grey areas = scotophase, white areas = photophase, yellow area = dim light exposure, green area = continuous food supply. The 11 series are detailed in Fig. 1B.

Figure 2 presents the actograms of the mean daily activity throughout the 97-day experiment, encompassing 11 series and the related results of chronobiological analyses based on individual daily activity illustrated in Figure S2. Two behavioral parameters were tested: valve opening amplitude (VOA, %) and valve opening duration (VOD, %). Under 12 L:12 D (series 1, 3, 5, 7, 9, 11), both VOA and VOD were synchronized by L: D exposure and exhibited a significant daily rhythm of ~ 24 h. The robustness of the rhythm, measured as PR (%) ranged between 49.6% and 63.4% (PR = 56.6 ± 2.3% mean ± SE) for VOA and between 41.0% and 57.2% (PR = 50.3 ± 2.3% mean ± SE) for VOD. To assess the plasticity of the daily rhythm, the series 4 displays the VOA and VOD activities, following a + 4 h-light shift. The results indicate a maintained significant daily rhythm of 23.6 ± 0.1 h (PR = 44.4%) for VOA and 23.8 ± 0.1 h (PR = 59.7%) for VOD. To investigate the endogenous circadian rhythm, mean behavioral activity was assessed under two free-running conditions: D:D and dim L: L. Under D: D (series 2), *O. edulis* demonstrated a significant rhythm within the circadian range (20–28 h) for VOA (25.8 ± 0.1 h) and for VOD (26.4 ± 0.1 h). The associated PR values were 44.2% for VOA and 40.7% for VOD. Under dim L: L (series 6), no significant circadian rhythms were detected for either VOA or VOD. Under ALAN exposure (series 8), a notable daily rhythm of 23.6 ± 0.1 h was observed for VOA, with a decreased PR (PR = 41.5%) in comparison to the L: D series. For VOD, no significant daily rhythm was noted. Although a significant periodicity (35.0 ± 0.1 h) was identified, using the Lomb & Scargle periodogram, the outcomes from the Cosinor model were not significant (*p* = 0.139). Finally, under feeding conditions (series 10), the results showed a significant daily rhythm of 23.9 ± 0,1 h (PR = 55.9%) for VOA and 24.0 ± 0.1 h (PR = 57.3%) for VOD.

### Individual chronobiological analyses of valve activity

**Fig. 3:**
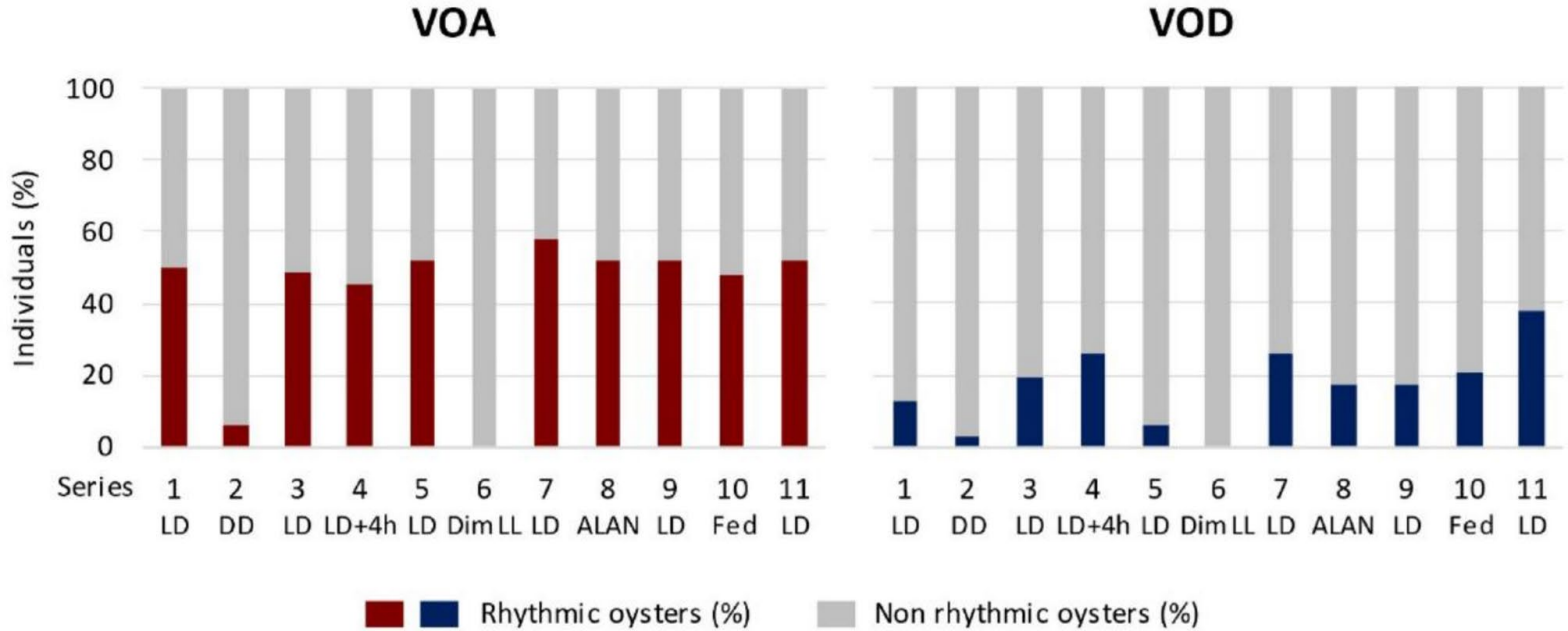
Individual chronobiological analyses of valve activity. Percentage of oysters with a significant daily rhythm for VOA (left panel) and VOD (right panel) during the 11 series (*n* = 16–32). The 11 series are detailed in Fig. 1B.

Figure 3 presents the results of individual chronobiological analyses of VOA and VOD across the 11 series, based on the data illustrated in Figure S2. Under 12 L:12 D (series 1, 3, 5, 7, 9, 11), the mean percentage of oysters exhibiting a significant daily rhythm was noticeably higher (*p* < 0.001, t-test) for VOA compared to VOD. For VOA, 52 ± 1% of oysters demonstrated a daily rhythm, with a minimum of 48% and a maximum of 58%. In contrast, for VOD, 20 ± 4% of oysters showed a daily rhythm, with a minimum of 6% and a maximum of 38%. In similar ranges, the percentages of rhythmic individuals during series 4 (L: D + 4 h), series 8 (ALAN), and series 10 (Fed) were 45%, 52% and 48% for VOA, and 26%, 17% and 21% for VOD, respectively. During the free-running D: D condition (series 2), the percentage of rhythmic oysters was only 6% for VOA and 3% for VOD (Fig. 3), attributed to prolonged closure events in a substantial number of individuals (Fig. S2). In the free-running dim L: L condition (series 6), no oysters exhibited a significant daily rhythm.

### Daily pattern of valve behavior

**Fig. 4:**
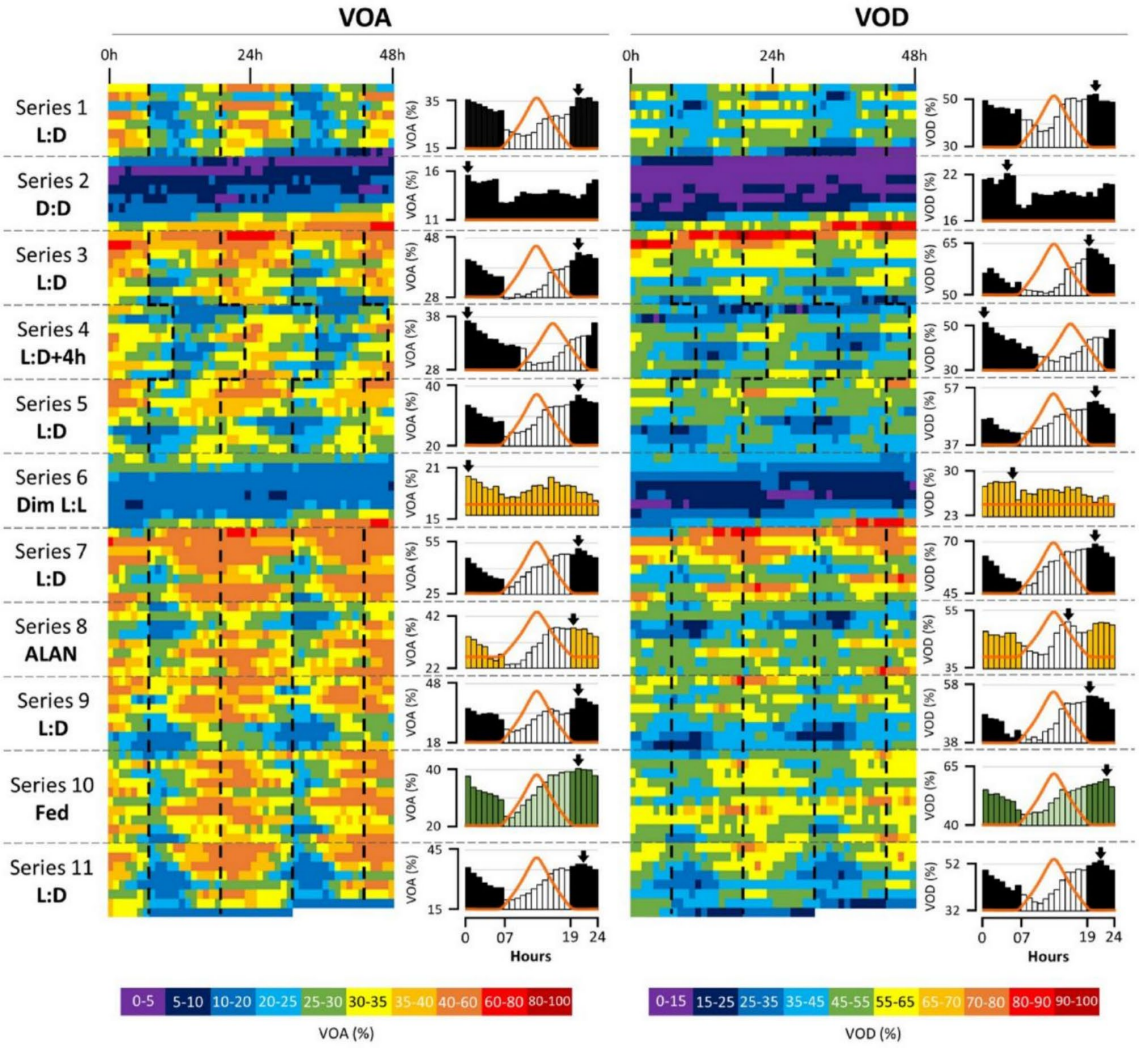
Daily pattern of *O. edulis* valve behavior. Heatmaps of mean VOA (left panel) and VOD (right panel) during the 11 series, 97 days in total (*n* = 16–32). For each heatmap of each series, a histogram of the corresponding mean daily pattern is associated. Black dotted line on the heatmaps separate scotophase and photophase. In histograms, dark bars = scotophase, white bars = photophase, yellow bars = dim light exposure, dark and light green bars = scotophase and photophase + feeding respectively. The black arrows show the daily peaks of activity. The orange lines represent the photoperiod for each series. The 11 series are detailed in Fig. 1B.

Figure 4 illustrate the mean daily patterns of the VOA and VOD during the 97-day experiment corresponding to 11 series, based on the individual daily pattern shown on Figure S3. For each behavioral parameters the results are shown as heatmaps associated with the corresponding mean daily patterns based on the average of the days of each series. Under 12 L:12 D (series 1, 3, 5, 7, 9, 11), both VOA and VOD showed a minimal activity just after transition to light phase (20–25% VOA and 35–45% VOD) and increased during the whole photophase to reach a peak of activity at the beginning of the night (40–60% VOA and 55–65% VOD). For all the series, VOA peak of activity was between 20 h and 22 h, i.e. 1–2 h after light off, while for VOD the peak of activity was between 19 h and 22 h, i.e. 0–2 h after light off. Individual daily VOA peak during L: D series are plotted in Figure S4 and showed a maximum of nocturnal peaks (75%). Under the + 4 h-light shift in series 4, both VOA and VOD nocturnal daily patterns were maintained and quickly synchronized by the shifted L: D regime, evidenced by the nocturnal peak of activity shifting by 4 h, i.e. still 1–2 h after light off. Under free-running conditions, mean daily VOA and VOD were significantly reduced in both D: D (series 2) and dim L: L (series 6). In D: D (series 2), the mean VOA peak of activity occurred at 0–1 h, reaching only ~ 16%, while the mean VOD peak was at 4–5 h, reaching only ~ 22%. In dim L: L (series 6), the mean VOA peak was also at 0–1 h, reaching only ~ 20%, whereas the mean VOD peak occurred at 5–6 h, reaching only ~ 28%. Under ALAN exposure (series 8), the peak activity of VOA was between 19 h and 20 h, i.e. 1–2 h earlier than the peak observed under the 12 L:12 D series, while the VOD peak occurred between 15 and 16 h, becoming diurnal, i.e. 4–7 h earlier than the peaks observed under 12 L:12 D. Under continuous feeding conditions, the VOA peak of activity remained between 20 h and 21 h, comparable to the peaks observed under the unfed 12 L:12 D series, while the VOD peak of activity was between 22 and 23 h, i.e. 1–3 h earlier than the peaks observed under the unfed 12 L:12 D series, and remained nocturnal. To complete and highlight individual nocturnal activity, the occurrence of daily peaks of VOA for the 32 individuals and the total for all individuals are detailed in Supplementary Fig. 4.

### Anticipation of valve activity diel behavior to daily light cycles

**Fig. 5:**
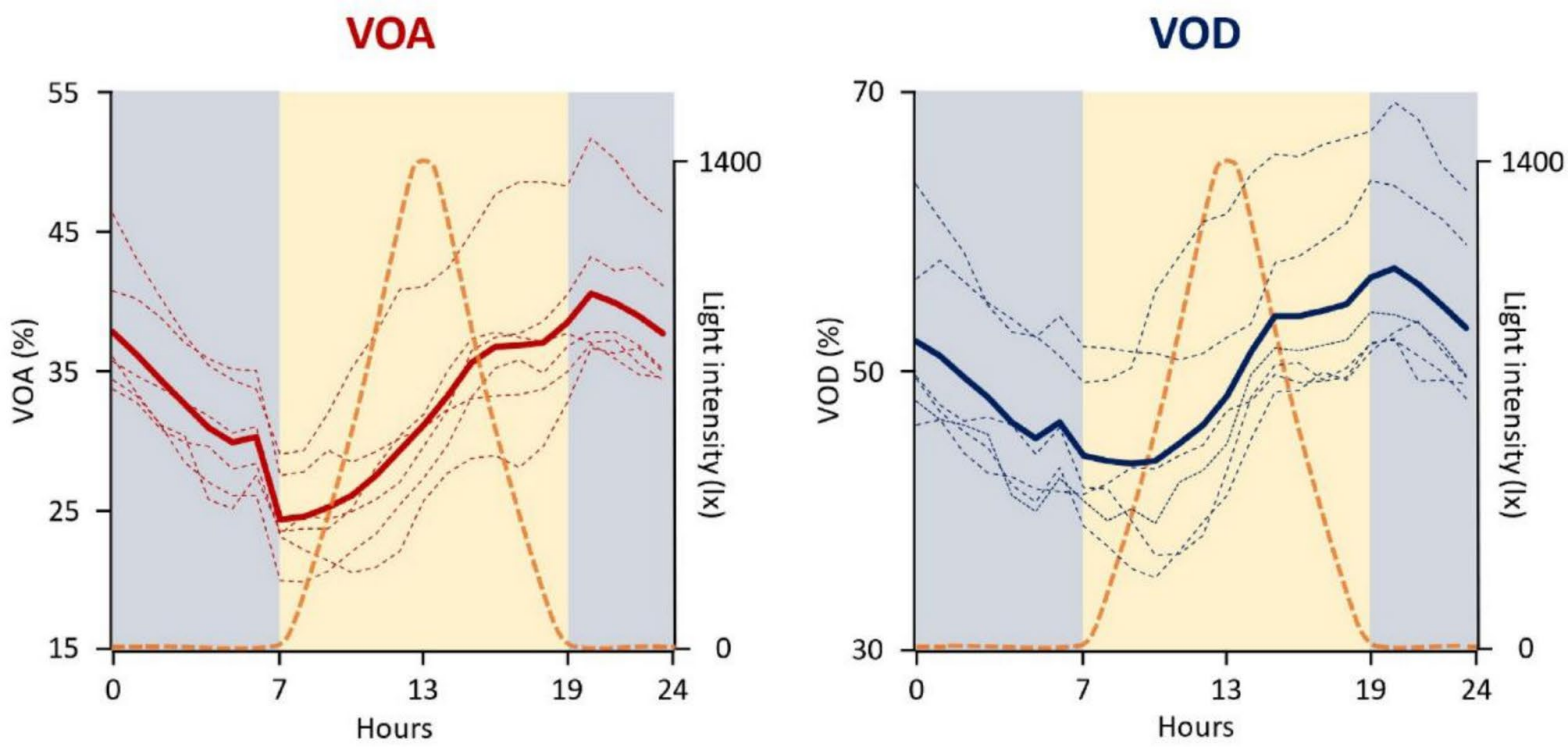
Anticipation of valve activity diel behavior to daily light cycles. Mean daily profile of mean VOA (left panel) and mean VOD (right panel) during the 6 references 12 L :12 D series (*n* = 16–32). In doted lines, the profiles of each series (*n* = 6 series, series 1, 3, 5, 7, 9, 11). In bold solid line, the mean profile of the 6 series. The dotted orange lines represent the photoperiod for the 6 references 12 L :12 D series.

To illustrate the anticipation of valve activity behavior in response to light cycle *zeitgebers*, Fig. 5 presents the mean daily profiles of mean VOA and mean VOD for the six reference 12 L :12 D series (series 1, 3, 5, 7, 9, 11). For both parameters, no direct response to light (switch on or switch off) was noted. A gradual decrease in activity commenced during the night, reaching a minimum in the early morning, followed by a gradual increase of activity that peaked in the early night. No direct correlation was identified between light intensity and valve behavior. To emphasize that the rhythmic behavior is not a direct response to light, Supplementary Fig. 5 displays the mean VOA and mean VOD profiles during the last day of series 1 (12 L :12 D) and the first two day of series 2 (D: D). The results indicate that during the second day of D: D, valve activity displays a similar rhythmic pattern to the L: D condition, despite a significant overall daily reduction in activity for both VOA and VOD.

## Discussion

This study demonstrates a synchronized daily valve activity in *O. edulis* for both VOA and VOD parameters under L: D conditions, characterized by a nocturnal peak of activity. With food supply, only the VOD acrophase is slightly altered but remains nocturnal. At the individual level, VOA displays more robust significant rhythms than VOD. Under free-running conditions (D: D), group-level results reveal a circadian rhythm, indicating an underlying endogenous clock mechanism. This assumption is reinforced by the anticipation of the light phase observed in the valve activity pattern under L: D and maintained during D: D. *O. edulis’* circadian clock can be described as labile and plastic due to its ability to adapt rapidly to a shift in light regime. Furthermore, even though a significant rhythm is found under D: D at the group level, at the individual level, only ~ 6% and ~ 3% of oysters are rhythmic for VOA and VOD, respectively. Under dim L: L condition, no circadian rhythm was observed, suggesting a masking effect despite the low light intensity applied. Lastly, under ALAN condition, the VOD daily rhythm disappears, and the peak of activity becomes diurnal.

### Characterization of *O. edulis* circadian clockwork

Typically, rhythms in marine organisms are described as robust under entrainment but labile, weak, or noisy in constant conditions^36–38^, which is confirmed for the native European oyster *O. edulis*. Although the daily rhythm is noted to be strong under L: D conditions, labile rhythmic oscillations were observed in free-running conditions where the rhythmicity is noisy and dampened at the individual level, indicating the presence of a functional circadian clock but with weak inertia. Gwinner and Brandstätter^38^ also describe a labile circadian oscillator as having a capacity for rapid adaptation to *zeitgeber* phase shifts^39,40^. This observation is further validated for *O. edulis* as resynchronization to a phase shift in the L: D cycle was immediate, without transient cycles.

The results also indicated that *O. edulis* does not respond directly to the daily light cycle but anticipates it, further attesting to an endogenous mechanism that optimizes its fitness^41,42^. Characterizing the robustness or the lability of the circadian clock could provide insights into the plasticity of the clock mechanism. A robust clock facilitates maximizing fitness in a stable cyclic environment. However, a clockwork that is too robust may be a limitation for the adaptation or expansion of the species, particularly for marine organisms. In coastal areas, numerous environmental cues can influence the behavior of resident species. Specifically, alongside solar day cycles, marine organisms are subject to tidal cycles, which typically occur twice per lunar day (24.8 h), approximately every 12.4 h. It is now well documented, that marine organisms can exhibit a strong tidal rhythm in addition to the daily rhythm^18,43,44^. The phase shift of the lunar day by ~ 51 min each solar day also results in a permanent phase shift between the two rhythms. Although recent findings at the molecular level suggest an interplay of molecular mechanisms involved in different rhythms^27^, a fundamental debate continues regarding the existence and nature of one or several core clocks to elucidate both concomitant circadian and circa-tidal rhythmicity^19^. Based on behavioral studies, three historical hypotheses have been proposed to explain the co-existence of tidal and daily rhythms. The first hypothesis, supported by Naylor^43^, posits the existence of two separate and unimodal circatidal and circadian clocks. The second hypothesis advocated by Palmer^45^, suggests the presence of two unimodal circalunidian clocks (24.8 h lunar day cycle) coupled in antiphase. Lastly, the third hypothesis originally advanced by Enright^46^, proposes a single clock that governs both circadian and circatidal rhythms. This hypothesis has received strongly backing from findings at both behavioral and molecular levels concerning the oyster *C. gigas*^19^ and has become increasingly accepted for various littoral species^27^. According to this hypothesis, a labile single clock would facilitate the adaptability to a phase shift between solar and lunar days and to modulate daily the bimodal pattern of behavior driven by both daily light: dark and tidal vibration cycles, the *zeitgebers* of this putative unique clock. Moreover, a labile/plastic unique clock would enable a marine species to adapt to the duration of tidal cycles that average 12.4 h, while not remaining equal to 12.4 h for each cycle due to modulation by local geographical (local biotope morphology and hydrodynamic) and meteorological (atmospheric pressure and wind) conditions. Thus, tides predominantly around 12.4 h, termed semi-diurnal tides, may, in certain parts of the world, oscillate at 24.8 h, referred to as diurnal tides, matching the lunar day period, and closely aligning with the daily period, necessitating considerable plasticity of the clockwork^43^. Similarly, the daily cycle, which exhibits less variability than that of the tidal one, predominantly remains around ~ 24 h, yet can vary significantly in terms of light phase duration, as observed in polar regions, where polar night (24 h of darkness) or polar day (24 h of light) can persist for months. In these areas, it has been demonstrated that the daily/circadian rhythms of bivalves are expressed, even during polar day or polar night, as seen in mussels *Mytilus sp*^22,32^ or in pecten *Chlamys islandica*^16^.

### What about a labile clock in an anthropized world exposed to global warming and the modification of the nocturnal Lightscape due to ALAN?

Endogenous clocks are adaptive mechanisms finely tuned over evolutionary timescales. They enable organisms to anticipate predictable changes in their environment and therefore adjust their physiology and behavior in expectation of these changes. Thus, by preparing their physiological processes, organisms can optimize cyclic opportunities (e.g. food supply, appropriate temperature for specific physiological processes, etc.) and mitigate cyclic risks (e.g. predation, UV radiation etc.) inherent to their environment^12^. However, the current rapid global climate change alters the temporal dynamics of biotic and abiotic environmental cycles to which endogenous clocks are adapted, while the daily light: dark cycle, i.e. the main *zeitgeber* for the circadian clock, is unaffected. Global warming could also induce latitudinal shifts in species distribution, as organisms track their optimal thermal environments toward the pole to avoid heat stress^47–50^. As the duration of the light phase in the daily light: dark cycle is specific to each latitude and time, organisms’ shift northward implies a new photic environment^51^. In both cases, whether there is a change in latitudinal or not, the proper alignment of endogenous rhythms and environmental cycles is challenged. The interpretation of the daily light signal by the endogenous clock must adapt to the new living environment to ensure the fitness of organisms. In this context, a labile clock, such as that described in *O. edulis*, may offer an adaptive advantage in coping with these rapid changes. Conversely, a clock that is too robust may increase the risk of a mismatch between organisms’ biological rhythms and their new environment. Notably, temporal mismatches between species in a food chain can jeopardize the survival of organisms^47,49,51^.

ALAN may also produce a risk of misalignment between endogenous rhythms and environmental cycles that disrupts the daily, seasonal, and lunar photic signals and hence could disrupt *zeitgebers* of endogenous clocks, constituting an unprecedented physical change over evolutionary time scales^29,52^. ALAN is currently widespread worldwide, with 83% of the world’s population living under a sky affected by ALAN^52,53^. Between 2011 and 2022, the sky’s brightness increased on average by 9.6% per year^54^. Among the surfaces exposed to ALAN, coastal areas are strongly impacted, and marine protected areas are not spared. Concerning the native European oyster *O. edulis’* habitats, i.e. Europe’s coastline areas, 54% are estimated to be light-polluted^30,55^. It is now a consensus that ALAN poses a severe threat to biodiversity and ecosystems, mainly because of the wrong photic signal it provides to biological clockworks^29,56–58^. In a context where daily light cycles are no longer reliable due to ALAN, processing a labile daily rhythm, as observed in *O. edulis*, which may be easily disrupted by photic cues, may disadvantage the organism in maintaining synchrony with its environment.

Consequently, as *O. edulis* is a sessile and benthic organism that cannot escape ALAN, a decrease in its fitness is highly anticipated in coastal shallow water environments. In this study, when oysters were exposed to a realistic and low level of ALAN (5 lx), no abolishment of the daily rhythm was observed, as seen under dim L: L. However, the results indicated a disappearance of the daily rhythm for VOD, with a shift of the maximum VOD from nocturnal to diurnal. These findings align with the hypothesis of the high susceptibility of *O. edulis* to ALAN, due to a clock characterized as labile. Such sensitivity to ALAN has recently been highlighted in its close congener, the oyster *C. gigas*, for which the circadian clock was also described as labile^17^. In this species, the behavioral daily rhythm, along with the expression of circadian clock and clock-associated genes, can be impaired by ALAN, even with a low exposure of 0.1 lx^31^. Moreover, it has been shown that the eyeless oyster *C. gigas* responds differently to ALAN depending on the light quality^59^ and exposure modality^60^.

This study contributes to filling a knowledge gap in understanding of the biology of *O. edulis* by characterizing the plasticity of its circadian clock. This adaptive (i.e. labile and plastic) clockwork may explain the wide historical distribution of *O. edulis* throughout the European waters from the Mediterranean Sea to Norway and from shallow coastal waters to depth of 80 m in sublittoral and offshore environments^1,4,5^. Today, over 85% of native oyster reef habitat is lost across Europe. The Native Oyster Restoration Alliance (NORA, https://noraeurope.eu/) supports knowledge exchange for the restoration and the protection of this endangered species, a key organism for biogenic reef habitats in European coastal ecosystems^1,3,4^. Light pollution should be considered as an increasing anthropogenic pressure, and a relevant parameter potentially affecting nearshore native oyster restoration measures. To our knowledge, this is the first study to assess the endogenous clock and behavioral rhythms in the native European oyster. Nevertheless, further analyses are needed to better characterize the circadian rhythm in terms of temperature compensation, which is a fundamental property of circadian rhythms. This property would inform us about this species adaptability in the context of climate change. Additionally, further chronobiological experiences are required to demonstrate that a functional circadian clock of *O. edulis* is entrainable by daily light dark cycles and responsible of apparent daily rhythms. The behavioral rhythms of this species remain to be studied in its natural environment to compare with laboratory results and to determine how *O. edulis* copes with the effects of climate change and anthropogenic pressures such as ALAN. Understanding of how environmental changes influence the behavioral rhythms of *O. edulis* (e.g., the combination of both natural and anthropogenic drivers) is a key point for succeeding in its recovery.

## Electronic supplementary material

Below is the link to the electronic supplementary material.

Supplementary Material 1

## Data Availability

The datasets used and analysed during the current study available from the corresponding author on reasonable request.
