# Supplementary material for: Characterization of the daily and circadian valve behavior of the European flat oyster Ostrea edulis
Source: Sci Rep. 2025 Apr 29;15:14952. doi: 10.1038/s41598-025-98746-x (PMC12041495; doi:10.1038/s41598-025-98746-x)
Supplement: Supplementary file 1 — Supplementary Material 1 [file 41598_2025_98746_MOESM1_ESM.pdf]

Supplementary Information

Characterization of the daily and circadian valve behavior of the European flat oyster *Ostrea edulis*

Alexandre LE MOAL. Damien TRAN. Laura PAYTON. Bernadette POGODA. Bettina MEYER

Journal: Scientific reports

Corresponding author: Alexandre Le Moal / Bettina Meyer

E-mail: alexandre.lemoal@awi.de / bettina.meyer@awi.de

The following items are provided:

Figure S1: Schematic principle of actogram and heatmap representations of mean valve opening amplitude (VOA) and mean valve opening duration (VOD).

Table S1: Individual chronobiological analyses of valve activity.

Figure S2: Actograms of *Ostrea edulis* valve activity at the individual level.

Figure S3: Daily pattern of *Ostrea edulis* valve behavior at the individual level.

Figure S4: Daily valve activity peak of *Ostrea edulis* under L:D regimes at individual level.

Figure S5: Transition of *Ostrea edulis* valve activity from L:D to D:D.

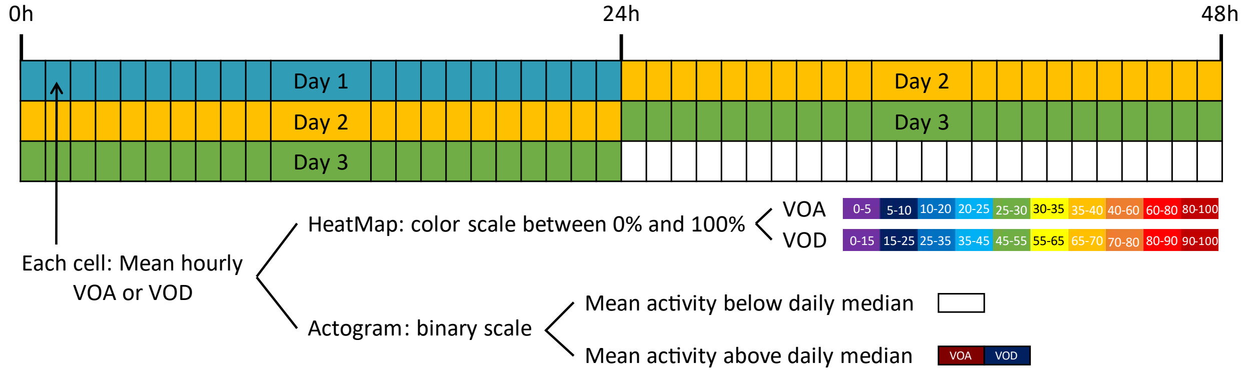

Figure S1. Schematic principle of actogram and heatmap representations of mean valve opening amplitude (VOA) and mean valve opening duration (VOD).

Table S1: Individual chronobiological analyses of valve activity. Results of the individual-chronobiological analysis for VOA and VOD for the two experiment units (EU1 and EU2). For each individual during each series is associated the characteristics of the Cosinor model: the period (h) ± SE and the *p-value*. "ns" represent a non-significant Cosinor model. Dark grey cells represent dead animals (or signal problem during series 1 for the EU2). The last line shows the mean individual period ± SE for each series.

	Series	VOA 1	VOA 2	VOA 3	VOA 4	VOA 5	VOA 6	VOA 7	VOA 8	VOA 9	VOA 10	VOA 11
EU 1	1	ns	ns	ns	ns	ns	ns	ns	ns	ns	ns	26.1 ± 0.07 p<0.001
	2	27.2 ± 0.08 p<0.001	ns	24.4 ± 0.07 p<0.001	23.6 ± 0.09 p<0.001	21.6 ± 0.09 p<0.001	ns	ns	ns	ns	ns	ns
	3	23.8 ± 0.08 p<0.001	ns	23.9 ± 0.08 p<0.001	26.9 ± 0.05 p<0.001	25.3 ± 0.08 p<0.001	ns	23.2 ± 0.07 p<0.001	22.5 ± 0.08 p<0.001	24.9 ± 0.06 p<0.001	ns	ns
	4	ns	26.0 ± 0.08 p<0.001	ns	ns	ns	ns	ns	27.4 ± 0.05 p<0.001	25.2 ± 0.06 p<0.001	ns	25.8 ± 0.05 p<0.001
	5	ns	ns	24.8 ± 0.05 p<0.001	ns	ns	ns	ns	Dead			
	6	23.0 ± 0.10 p<0.001	ns	ns	23.6 ± 0.09 p<0.001	22.1 ± 0.07 p<0.001	ns	ns	ns	ns	22.3 ± 0.12 p<0.001	ns
	7	ns	24.3 ± 0.07 p<0.001	ns	22.8 ± 0.08 p<0.001	22.4 ± 0.09 p<0.001	ns	ns	ns	23.7 ± 0.09 p<0.001	23.8 ± 0.08 p<0.001	23.5 ± 0.07 p<0.001
	8	24.0 ± 0.05 p<0.001	ns	23.6 ± 0.05 p<0.001	ns	ns	ns	ns	27.4 ± 0.06 p<0.001	ns	ns	ns
	9	26.4 ± 0.06 p<0.001	ns	ns	ns	ns	ns	23.9 ± 0.06 p<0.001	22.9 ± 0.06 p<0.001	23.6 ± 0.06 p<0.001	20.9 ± 0.09 p<0.001	26.1 ± 0.05 p<0.001
	10	22.1 ± 0.09 p<0.001	ns	26.2 ± 0.05 p<0.001	ns	ns	ns	24.0 ± 0.07 p<0.001	ns	ns	ns	27.6 ± 0.06 p<0.001
	11	27.0 ± 0.08 p<0.001	ns	ns	ns	ns	ns	25.6 ± 0.07 p<0.001	25.3 ± 0.05 p<0.001	ns	23.7 ± 0.06 p<0.001	ns
	12	25.1 ± 0.08 p<0.001	ns	ns	ns	ns	ns	ns	ns	ns	ns	24.1 ± 0.05 p<0.001
	13	ns	ns	ns	ns	ns	ns	23.5 ± 0.08 p<0.001	ns	22.9 ± 0.07 p<0.001	23.6 ± 0.09 p<0.001	22.5 ± 0.09 p<0.001
	14	ns	ns	20.0 ± 0.12 p<0.001	23.7 ± 0.06 p<0.001	24.2 ± 0.08 p<0.001	ns	24.6 ± 0.05 p<0.001	22.8 ± 0.07 p<0.001	23.0 ± 0.07 p<0.001	24.0 ± 0.06 p<0.001	25.1 ± 0.08 p<0.001
	15	ns	ns	ns	24.2 ± 0.05 p<0.001	24.6 ± 0.06 p<0.001	ns	22.4 ± 0.05 p<0.001	23.4 ± 0.08 p<0.001	ns	ns	ns
	16	ns	ns	ns	ns	22.6 ± 0.07 p<0.001	ns	ns	25.5 ± 0.05 p<0.001	ns	ns	ns
EU 2	1	Signal problem	ns	22.6 ± 0.09 p<0.001	22.6 ± 0.07 p<0.001	ns	ns	24.0 ± 0.08 p<0.001	ns	ns	ns	ns
	2		ns	27.2 ± 0.08 p<0.001	24.4 ± 0.05 p<0.001	25.4 ± 0.06 p<0.001	ns	ns	27.7 ± 0.06 p<0.001	25.6 ± 0.04 p<0.001	24.2 ± 0.07 p<0.001	23.6 ± 0.09 p<0.001
	3		ns	24.1 ± 0.07 p<0.001	22.5 ± 0.08 p<0.001	ns	ns	ns	Dead			
	4		ns	ns	24.3 ± 0.05 p<0.001	ns	ns	25.1 ± 0.08 p<0.001	ns	26.1 ± 0.07 p<0.001	23.4 ± 0.09 p<0.001	ns
	5		ns	24.2 ± 0.05 p<0.001	24.1 ± 0.06 p<0.001	24.0 ± 0.09 p<0.001	ns	23.6 ± 0.08 p<0.001	ns	24.8 ± 0.07 p<0.001	ns	25.5 ± 0.05 p<0.001
	6		ns	Dead								
	7		ns	ns	ns	ns	ns	ns	ns	25.7 ± 0.08 p<0.001	24.3 ± 0.08 p<0.001	ns
	8		ns	ns	ns	22.6 ± 0.08 p<0.001	ns	24.0 ± 0.07 p<0.001	ns	ns	24.6 ± 0.10 p<0.001	26.3 ± 0.07 p<0.001
	9		ns	23.0 ± 0.08 p<0.001	ns	ns	ns	21.7 ± 0.09 p<0.001	27.2 ± 0.03 p<0.001	21.0 ± 0.11 p<0.001	ns	24.2 ± 0.04 p<0.001
	10		ns	ns	ns	23.4 ± 0.09 p<0.001	ns	25.1 ± 0.06 p<0.001	24.5 ± 0.07 p<0.001	23.6 ± 0.09 p<0.001	23.6 ± 0.09 p<0.001	ns
	11		ns	24.3 ± 0.04 p<0.001	ns	24.1 ± 0.05 p<0.001	ns	25.5 ± 0.04 p<0.001	25.1 ± 0.09 p<0.001	22.9 ± 0.06 p<0.001	23.5 ± 0.07 p<0.001	24.2 ± 0.04 p<0.001
	12		ns	24.2 ± 0.06 p<0.001	ns	23.1 ± 0.07 p<0.001	ns	24.0 ± 0.06 p<0.001	24.1 ± 0.07 p<0.001	24.2 ± 0.07 p<0.001	27.6 ± 0.05 p<0.001	23.0 ± 0.08 p<0.001
	13		ns	24.4 ± 0.05 p<0.001	ns	ns	ns	24.8 ± 0.07 p<0.001	ns	ns	21.7 ± 0.08 p<0.001	ns
	14		ns	20.8 ± 0.11 p<0.001	24.4 ± 0.08 p<0.001	25.7 ± 0.08 p<0.001	ns	25.4 ± 0.06 p<0.001	ns	ns	ns	ns
	15		ns	ns	22.3 ± 0.05 p<0.001	24.5 ± 0.06 p<0.001	ns	25.2 ± 0.07 p<0.001	22.6 ± 0.08 p<0.001	23.8 ± 0.09 0.0001	ns	26.9 ± 0.06 p<0.001
	16		ns	ns	23.8 ± 0.06 p<0.001	23.7 ± 0.07 p<0.001	ns	ns	23.7 ± 0.08 p<0.001	ns	Dead	
Mean		24.5 ± 0.87	25.1 ± 0.85	23.8 ± 0.46	23.8 ± 0.31	23.7 ± 0.31	/	24.4 ± 0.32	24.8 ± 0.49	24.1 ± 0.35	23.6 ± 0.41	24.9 ± 0.39

	Series	VOD 1	VOD 2	VOD 3	VOD 4	VOD 5	VOD 6	VOD 7	VOD 8	VOD 9	VOD 10	VOD 11
EU 1	1	ns	ns	ns	ns	ns	ns	ns	ns	ns	ns	26.3 ± 0.07 p<0.001
	2	ns	ns	ns	ns	ns	ns	ns	24.4 ± 0.09 p<0.001	ns	ns	27.6 ± 0.06 p<0.001
	3	ns	ns	ns	25.8 ± 0.06 p<0.001	ns	ns	ns	ns	ns	ns	ns
	4	ns	26.6 ± 0.07 p<0.001	ns	ns	ns	ns	ns	27.6 ± 0.07 p<0.001	25.5 ± 0.07 p<0.001	22.0 ± 0.09 p<0.001	25.7 ± 0.06 p<0.001
	5	ns	ns	24.3 ± 0.08 p<0.001	ns	ns	ns	ns	Dead			
	6	ns	ns	ns	ns	ns	ns	ns	ns	ns	ns	ns
	7	ns	ns	ns	22.6 ± 0.09 p<0.001	ns	ns	ns	ns	ns	ns	23.8 ± 0.08 p<0.001
	8	23.5 ± 0.09 p<0.001	ns	ns	ns	ns	ns	ns	ns	ns	ns	ns
	9	ns	ns	ns	ns	ns	ns	ns	ns	23.4 ± 0.07 p<0.001	ns	25.2 ± 0.07 p<0.001
	10	21.8 ± 0.11 p<0.001	ns	26.9 ± 0.07 p<0.001	ns	ns	ns	ns	ns	ns	ns	28.0 ± 0.07 p<0.001
	11	ns	ns	ns	24.3 ± 0.09 p<0.001	ns	ns	25.2 ± 0.06 p<0.001	ns	ns	ns	ns
	12	ns	ns	25.7 ± 0.08 p<0.001	22.0 ± 0.10 p<0.001	ns	ns	ns	ns	ns	ns	24.2 ± 0.08 p<0.001
	13	ns	ns	ns	ns	ns	ns	26.2 ± 0.08 p<0.001	26.9 ± 0.07 p<0.001	23.7 ± 0.06 p<0.001	23.6 ± 0.11 p<0.001	ns
	14	ns	ns	ns	ns	23.9 ± 0.07 p<0.001	ns	25.0 ± 0.05 p<0.001	ns	ns	ns	ns
	15	ns	ns	ns	ns	ns	ns	ns	ns	ns	ns	ns
	16	ns	ns	ns	ns	ns	ns	ns	ns	ns	ns	ns
EU 2	1	Signal problem	ns	ns	ns	ns	ns	ns	ns	ns	ns	ns
	2		ns	ns	24.4 ± 0.05 p<0.001	ns	ns	ns	ns	25.6 ± 0.07 p<0.001	ns	ns
	3		ns	ns	ns	ns	ns	25.2 ± 0.07 p<0.001	Dead			
	4		ns	ns	ns	ns	ns	ns	ns	ns	ns	ns
	5		ns	24.2 ± 0.05 p<0.001	23.7 ± 0.05 p<0.001	ns	ns	ns	ns	ns	24.1 ± 0.09 p<0.001	ns
	6		ns	Dead								
	7		ns	ns	ns	ns	ns	ns	27.4 ± 0.06 p<0.001	ns	ns	ns
	8		ns	ns	ns	ns	ns	23.9 ± 0.08 p<0.001	ns	ns	ns	ns
	9		ns	ns	ns	ns	ns	ns	ns	ns	25.5 ± 0.08 p<0.001	23.6 ± 0.08 p<0.001
	10		ns	ns	ns	ns	ns	ns	ns	ns	ns	ns
	11		ns	24.2 ± 0.07 p<0.001	ns	20.1 ± 0.09 p<0.001	ns	25.6 ± 0.06 p<0.001	ns	27.4 ± 0.05 p<0.001	ns	24.3 ± 0.08 p<0.001
	12		ns	ns	25.6 ± 0.06 p<0.001	ns	ns	23.6 ± 0.07 p<0.001	ns	ns	21.6 ± 0.12 p<0.001	23.4 ± 0.10 p<0.001
	13		ns	ns	ns	ns	ns	ns	ns	ns	22.1 ± 0.11 p<0.001	ns
	14		ns	24.8 ± 0.08 p<0.001	ns	ns	ns	25.1 ± 0.08 p<0.001	ns	ns	ns	ns
	15		ns	ns	ns	ns	ns	ns	ns	ns	ns	25.8 ± 0.07 p<0.001
	16		ns	ns	24.6 ± 0.07 p<0.001	23.6 ± 0.08 p<0.001	ns	ns	23.6 ± 0.07 p<0.001	ns	Dead	
Mean		22.6 ± 0.85	26.6 ± 0.00	25.0 ± 0.44	24.1 ± 0.47	22.5 ± 1.22	/	25.0 ± 0.30	26.0 ± 0.83	25.1 ± 0.73	23.1 ± 0.62	25.3 ± 0.78

A1

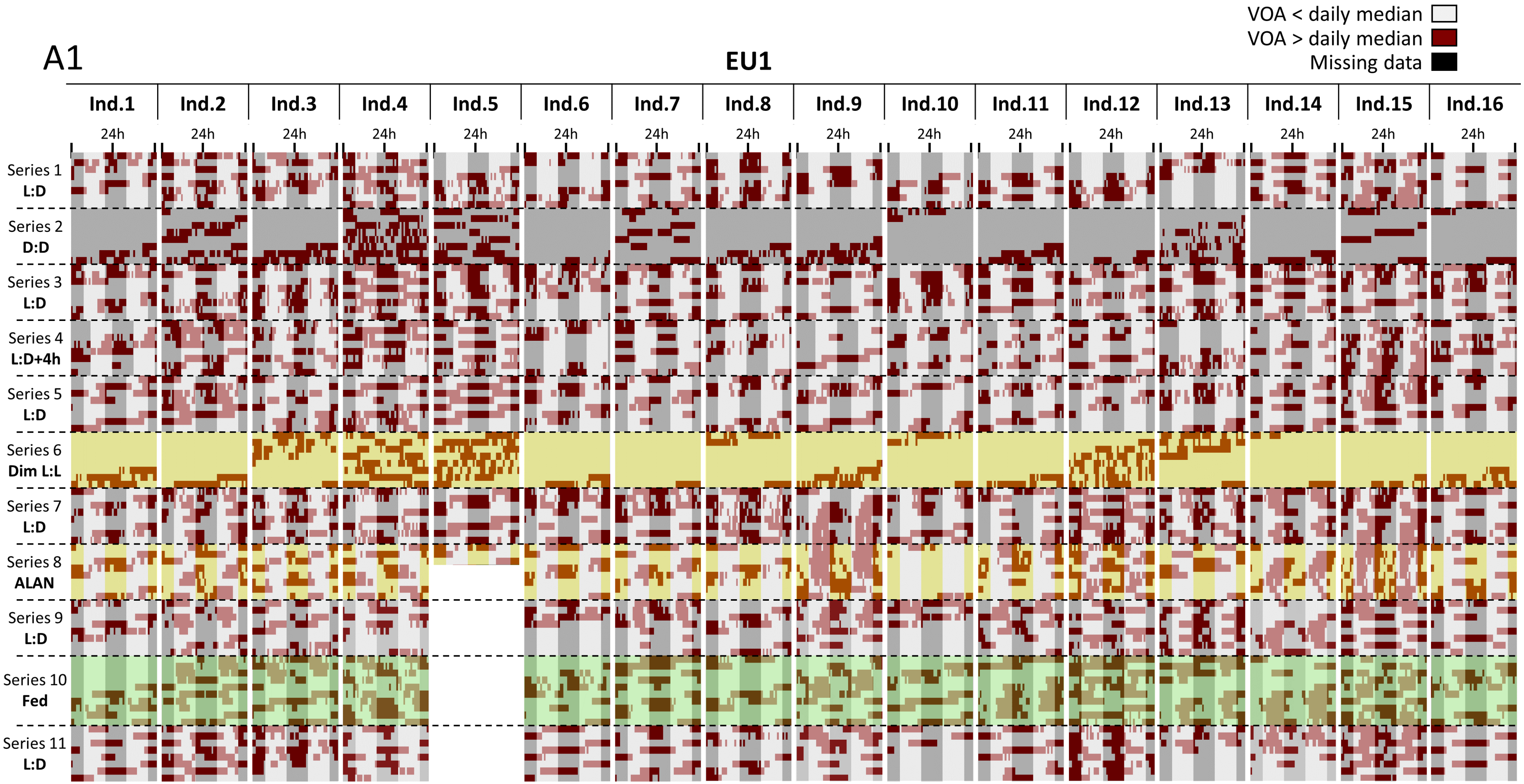
VOA < daily median
VOA > daily median
Missing data
EU1
Ind.1
Ind.2
Ind.3
Ind.4
Ind.5
Ind.6
Ind.7
Ind.8
Ind.9
Ind.10
Ind.11
Ind.12
Ind.13
Ind.14
Ind.15
Ind.16
24h
Series 1 L:D
Series 2 D:D
Series 3 L:D
Series 4 L:D+4h
Series 5 L:D
Series 6 Dim L:L
Series 7 L:D
Series 8 ALAN
Series 9 L:D
Series 10 Fed
Series 11 L:D

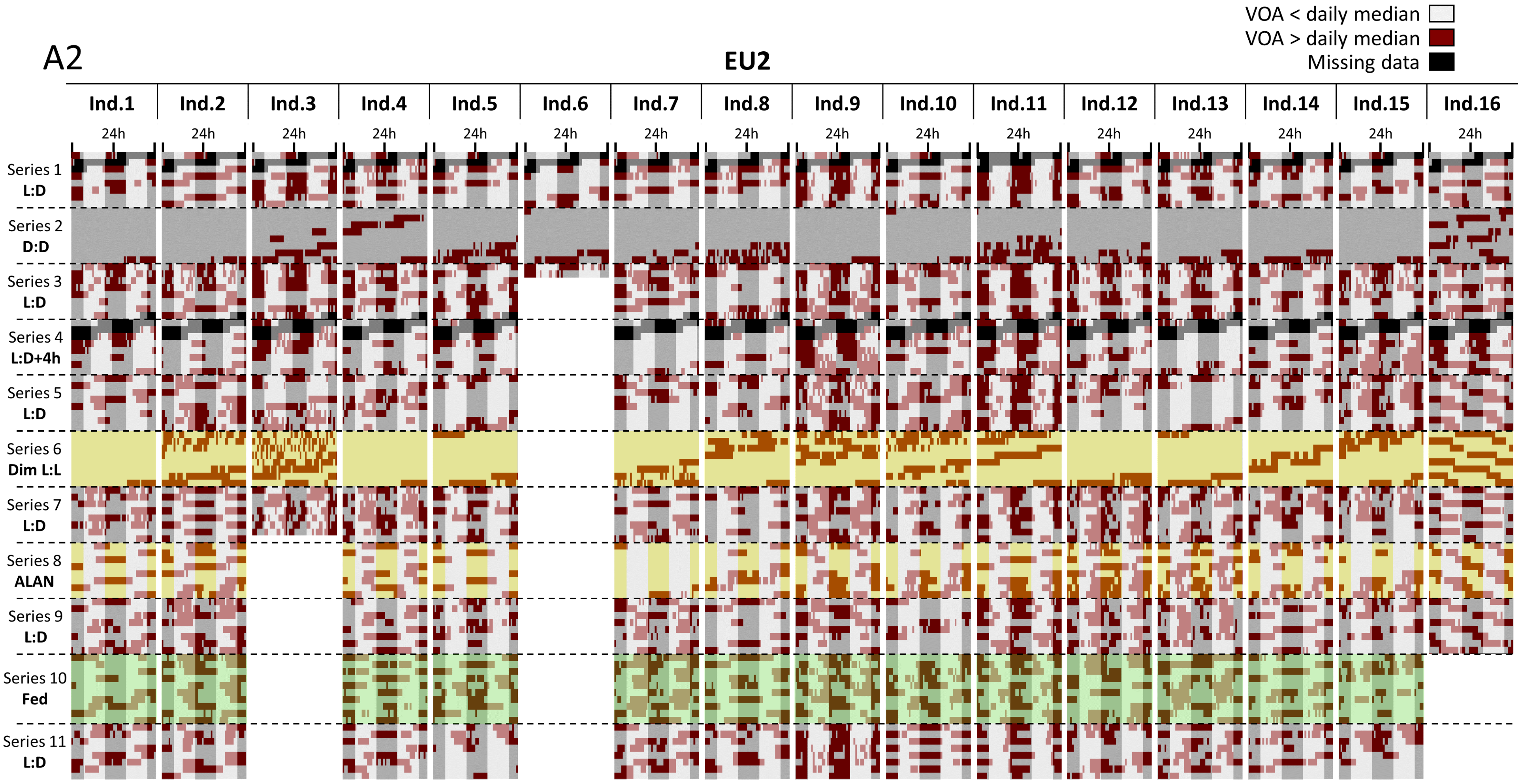
A2
EU2
VOA < daily median
VOA > daily median
Missing data
Ind.1
Ind.2
Ind.3
Ind.4
Ind.5
Ind.6
Ind.7
Ind.8
Ind.9
Ind.10
Ind.11
Ind.12
Ind.13
Ind.14
Ind.15
Ind.16
24h
Series 1 L:D
Series 2 D:D
Series 3 L:D
Series 4 L:D+4h
Series 5 L:D
Series 6 Dim L:L
Series 7 L:D
Series 8 ALAN
Series 9 L:D
Series 10 Fed
Series 11 L:D

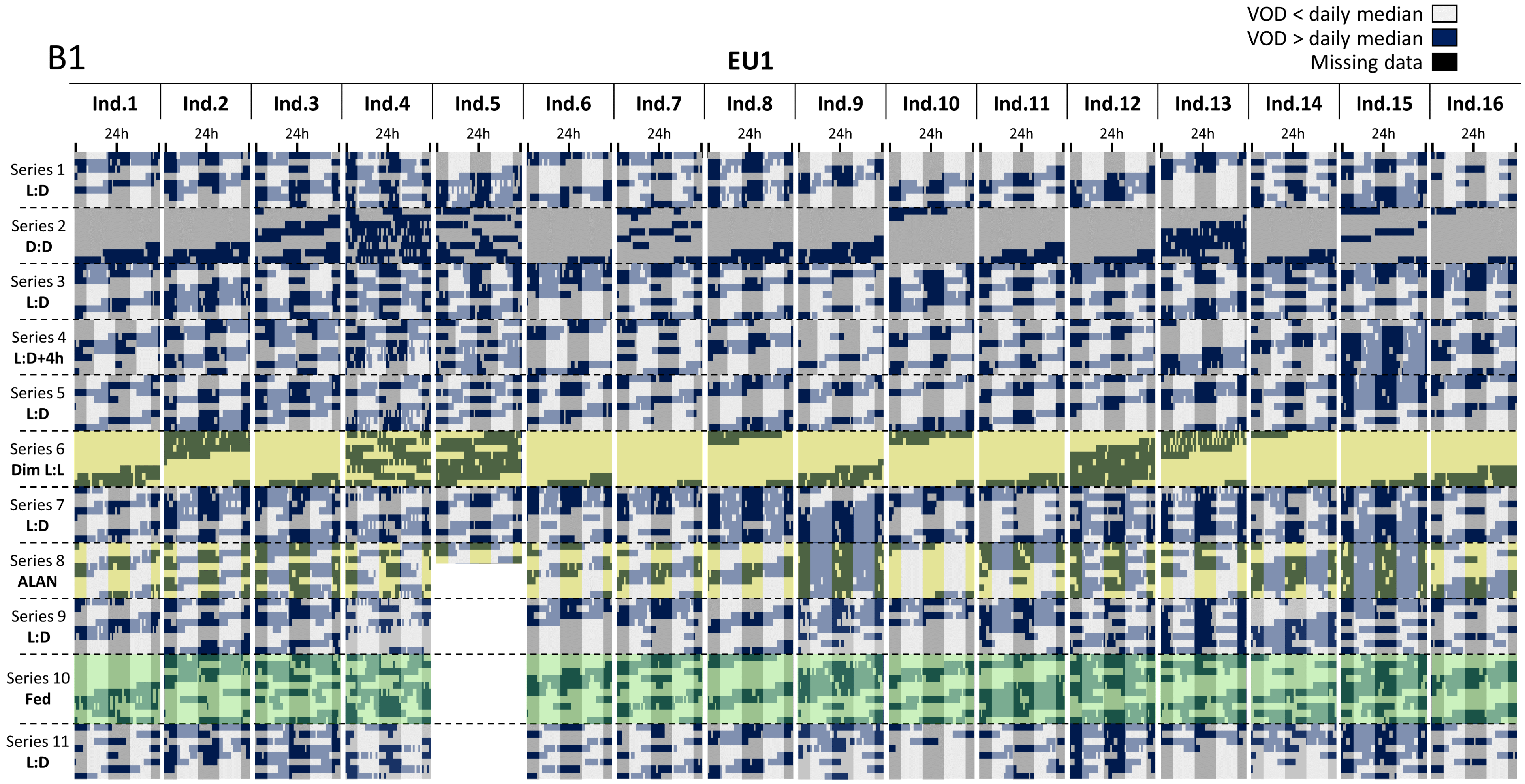
B1
EU1
VOD < daily median
VOD > daily median
Missing data
Ind.1
Ind.2
Ind.3
Ind.4
Ind.5
Ind.6
Ind.7
Ind.8
Ind.9
Ind.10
Ind.11
Ind.12
Ind.13
Ind.14
Ind.15
Ind.16
24h
Series 1 L:D
Series 2 D:D
Series 3 L:D
Series 4 L:D+4h
Series 5 L:D
Series 6 Dim L:L
Series 7 L:D
Series 8 ALAN
Series 9 L:D
Series 10 Fed
Series 11 L:D

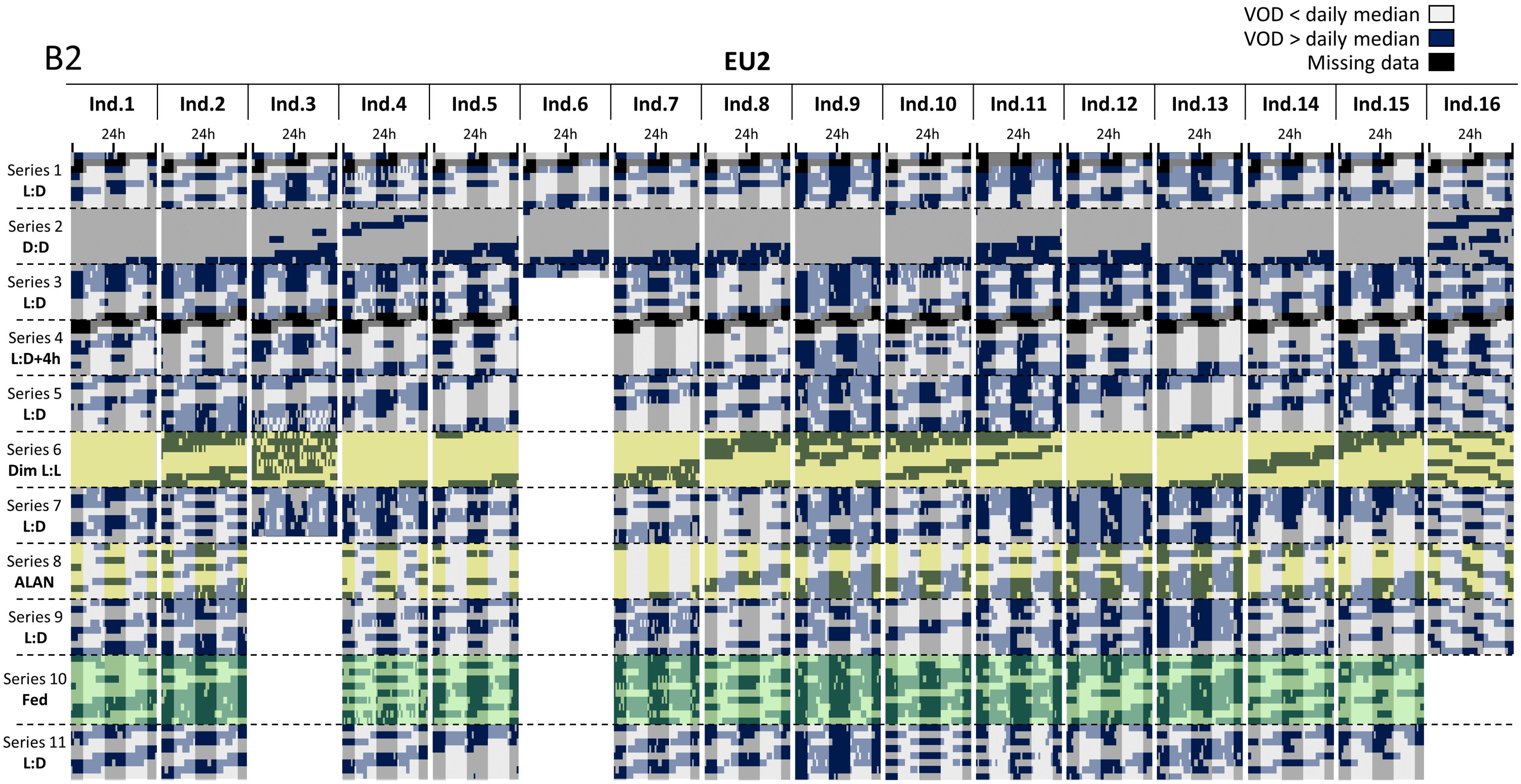

Figure S2. Actograms of *Ostrea edulis* valve activity at the individual level. Actograms of VOA (A1-A2) and VOD (B1-B2) during the 11 series, 97 days in total for the 32 individuals divided in 2 experimental units (EU1 and EU2). On actograms, dark grey areas = scotophase, white areas = photophase, yellow area = dim light exposure, green area = continuous food supply. The 11 series are detailed in Fig. 1B.

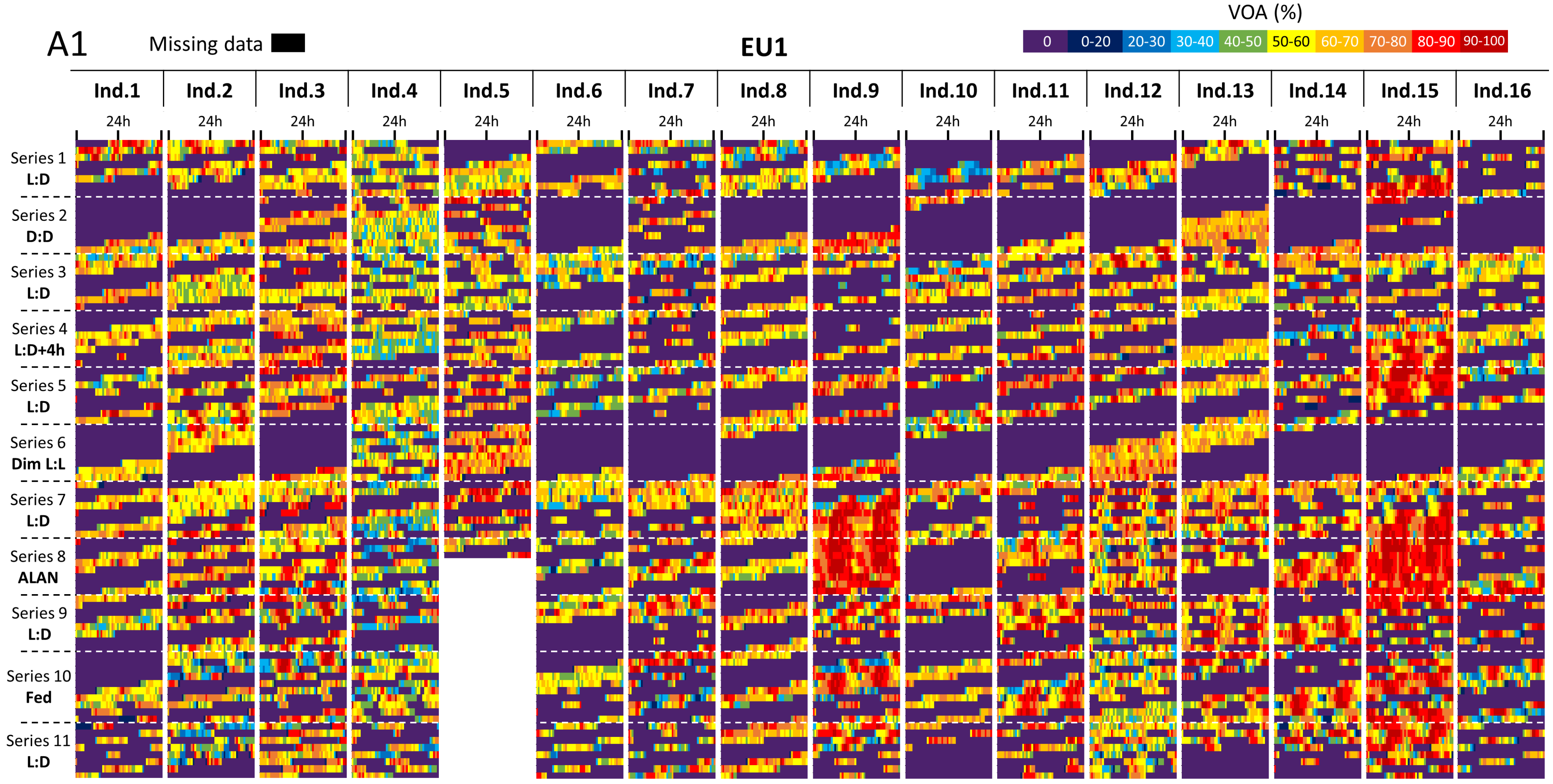
A1
Missing data
EU1
VOA (%)
0
0-20
20-30
30-40
40-50
50-60
60-70
70-80
80-90
90-100
Ind.1
Ind.2
Ind.3
Ind.4
Ind.5
Ind.6
Ind.7
Ind.8
Ind.9
Ind.10
Ind.11
Ind.12
Ind.13
Ind.14
Ind.15
Ind.16
24h
Series 1 L:D
Series 2 D:D
Series 3 L:D
Series 4 L:D+4h
Series 5 L:D
Series 6 Dim L:L
Series 7 L:D
Series 8 ALAN
Series 9 L:D
Series 10 Fed
Series 11 L:D

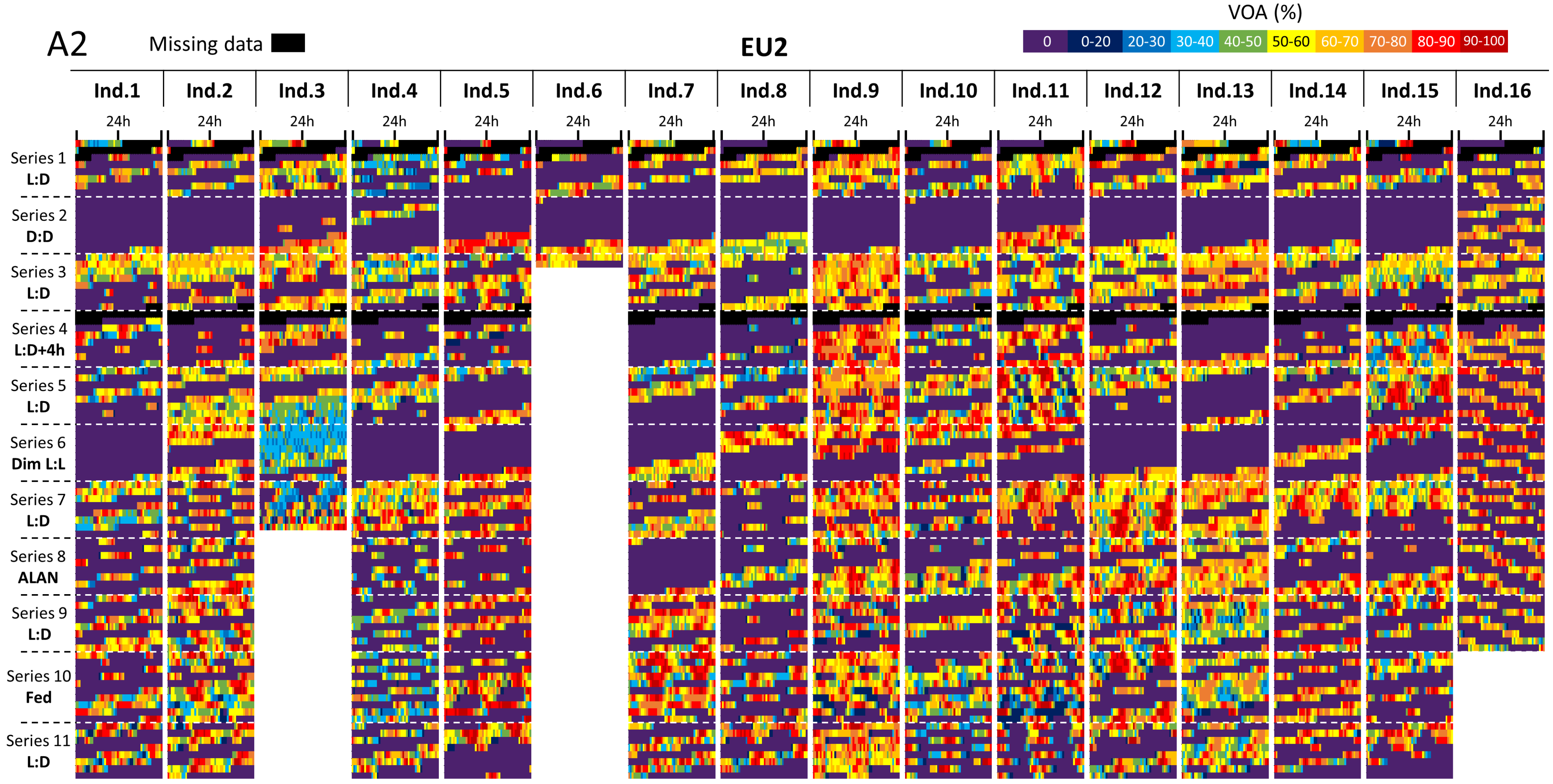
A2
Missing data
EU2
VOA (%)
0
0-20
20-30
30-40
40-50
50-60
60-70
70-80
80-90
90-100
Ind.1
Ind.2
Ind.3
Ind.4
Ind.5
Ind.6
Ind.7
Ind.8
Ind.9
Ind.10
Ind.11
Ind.12
Ind.13
Ind.14
Ind.15
Ind.16
24h
Series 1
L:D
Series 2
D:D
Series 3
L:D
Series 4
L:D+4h
Series 5
L:D
Series 6
Dim L:L
Series 7
L:D
Series 8
ALAN
Series 9
L:D
Series 10
Fed
Series 11
L:D

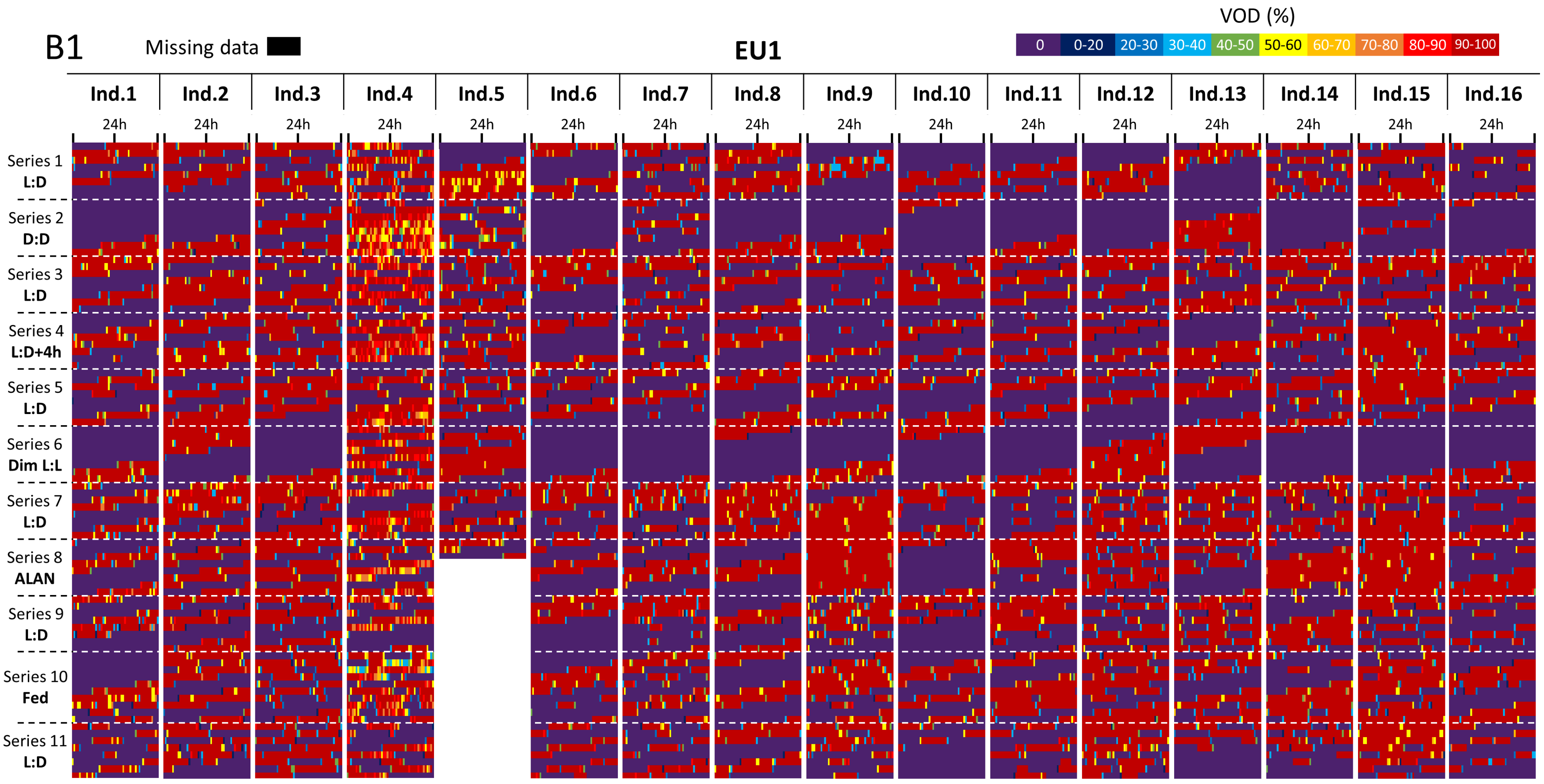
B1
Missing data
EU1
VOD (%)
0
0-20
20-30
30-40
40-50
50-60
60-70
70-80
80-90
90-100
Ind.1
Ind.2
Ind.3
Ind.4
Ind.5
Ind.6
Ind.7
Ind.8
Ind.9
Ind.10
Ind.11
Ind.12
Ind.13
Ind.14
Ind.15
Ind.16
24h
Series 1 L:D
Series 2 D:D
Series 3 L:D
Series 4 L:D+4h
Series 5 L:D
Series 6 Dim L:L
Series 7 L:D
Series 8 ALAN
Series 9 L:D
Series 10 Fed
Series 11 L:D

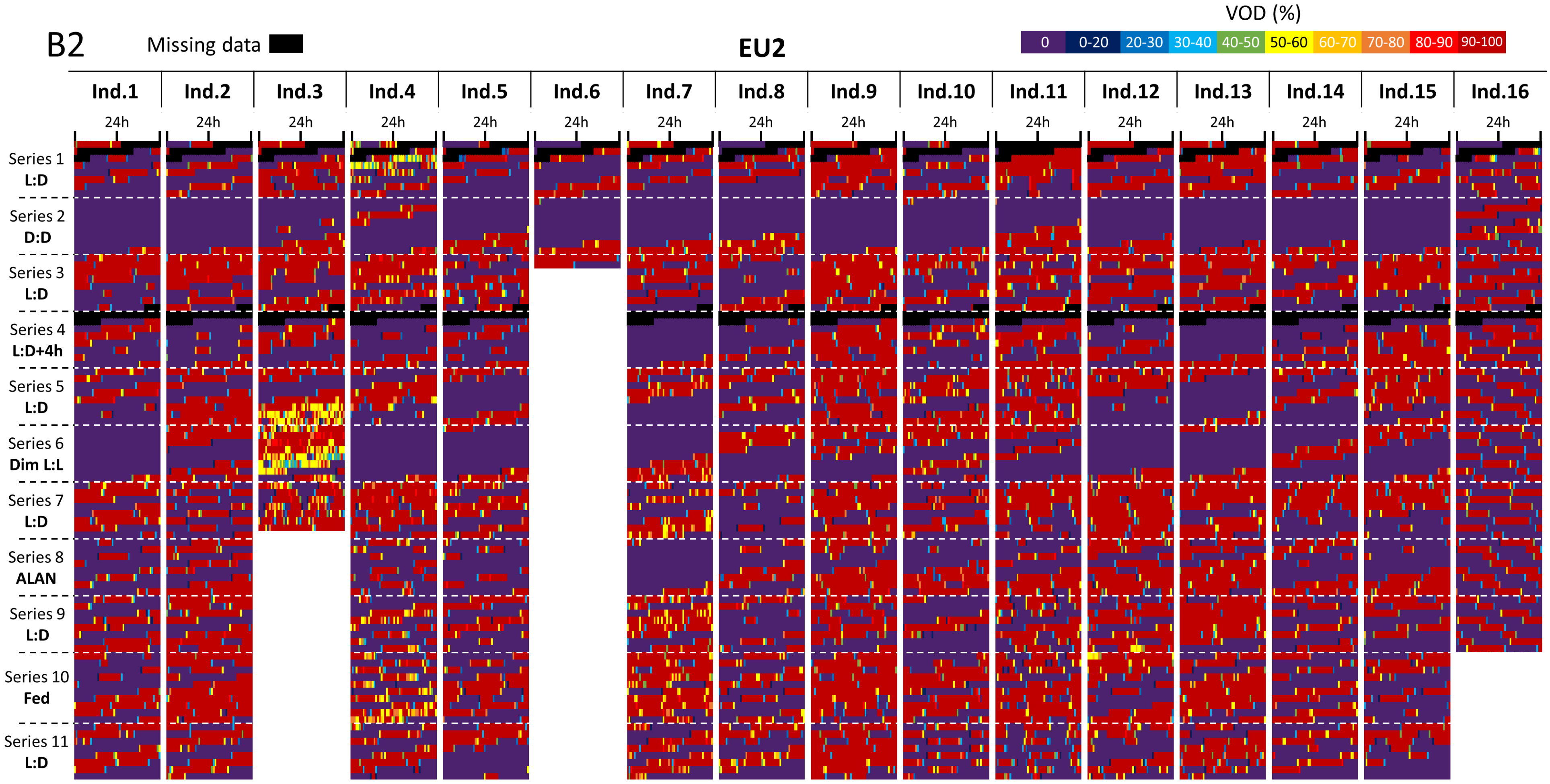

Figure S3. Daily pattern of *Ostrea edulis* valve behavior at the individual level. Heatmaps of VOA (A1-A2) and VOD (B1-B2) during the 11 series, 97 days in total for the 32 individuals divided in 2 experimental units (EU1 and EU2). The 11 series are detailed in Fig. 1B.

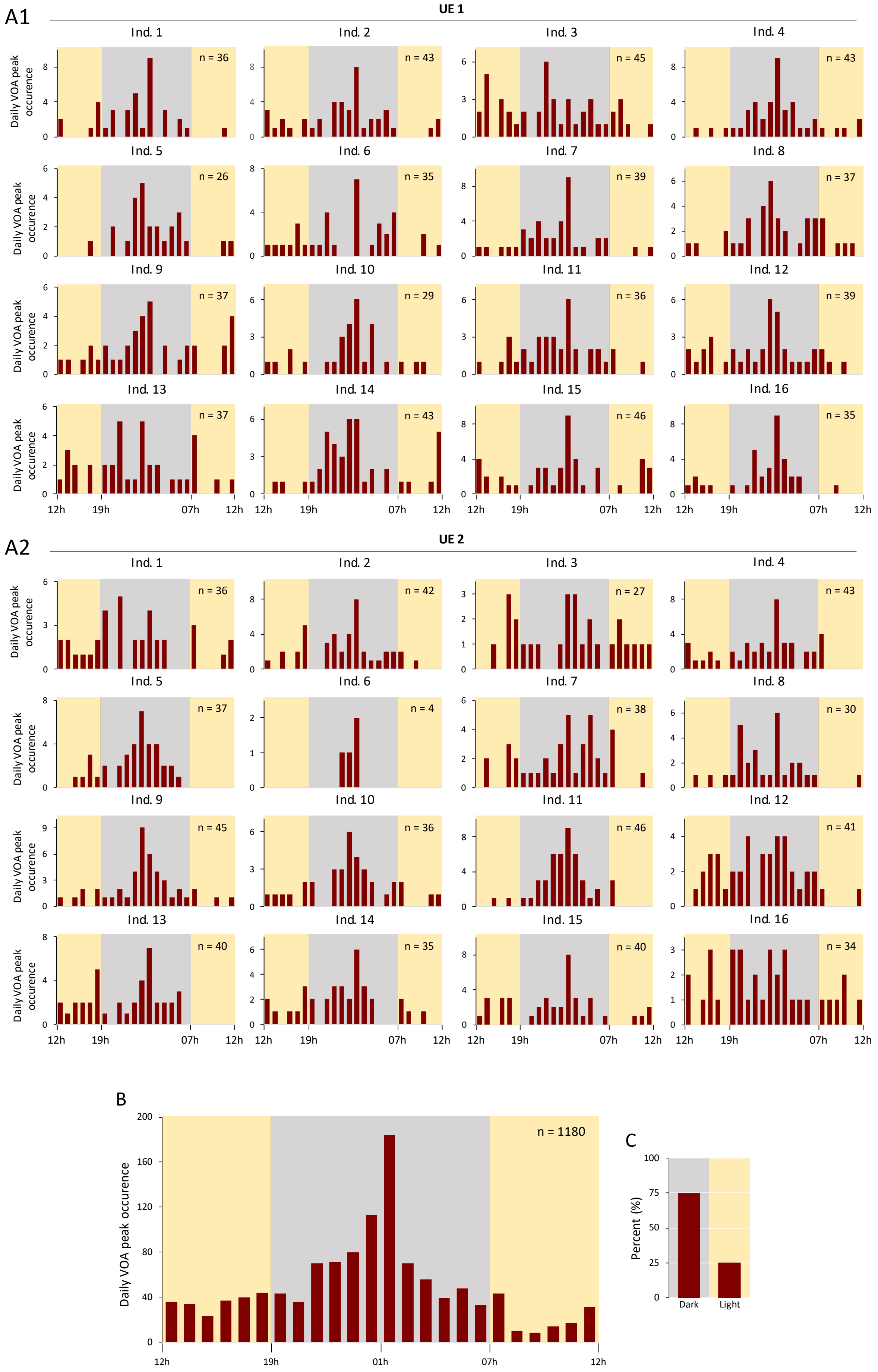
A1
UE 1
Ind. 1
Ind. 2
Ind. 3
Ind. 4
n = 36
n = 43
n = 45
n = 43
Ind. 5
Ind. 6
Ind. 7
Ind. 8
n = 26
n = 35
n = 39
n = 37
Ind. 9
Ind. 10
Ind. 11
Ind. 12
n = 37
n = 29
n = 36
n = 39
Ind. 13
Ind. 14
Ind. 15
Ind. 16
n = 37
n = 43
n = 46
n = 35
Daily VOA peak occurence
12h
19h
07h
12h
A2
UE 2
Ind. 1
Ind. 2
Ind. 3
Ind. 4
n = 36
n = 42
n = 27
n = 43
Ind. 5
Ind. 6
Ind. 7
Ind. 8
n = 37
n = 4
n = 38
n = 30
Ind. 9
Ind. 10
Ind. 11
Ind. 12
n = 45
n = 36
n = 46
n = 41
Ind. 13
Ind. 14
Ind. 15
Ind. 16
n = 40
n = 35
n = 40
n = 34
B
n = 1180
Daily VOA peak occurence
12h
19h
01h
07h
12h
C
Percent (%)
Dark
Light

Figure S4. Daily valve activity peak of *Ostrea edulis* under L:D regimes at individual level. (A1-A2) Histograms of daily VOA peak occurrences during L:D regimes (series 1, 3, 5, 7, 9, and 11) for the 32 individuals divided in 2 experimental units (EU1 and EU2). (B) Histograms of daily VOA peak occurrences during L:D regimes for all the individuals (n= 32). (C) Percentage of daily VOA peaks during scotophase and photophase for all individuals (n = 32) during all L:D series. Dark grey areas represent scotophase, yellow areas represent photophase. "n" in each graph indicates the number of days taken into account, i.e. the number of daily peaks. Days of continuous closure are excluded.

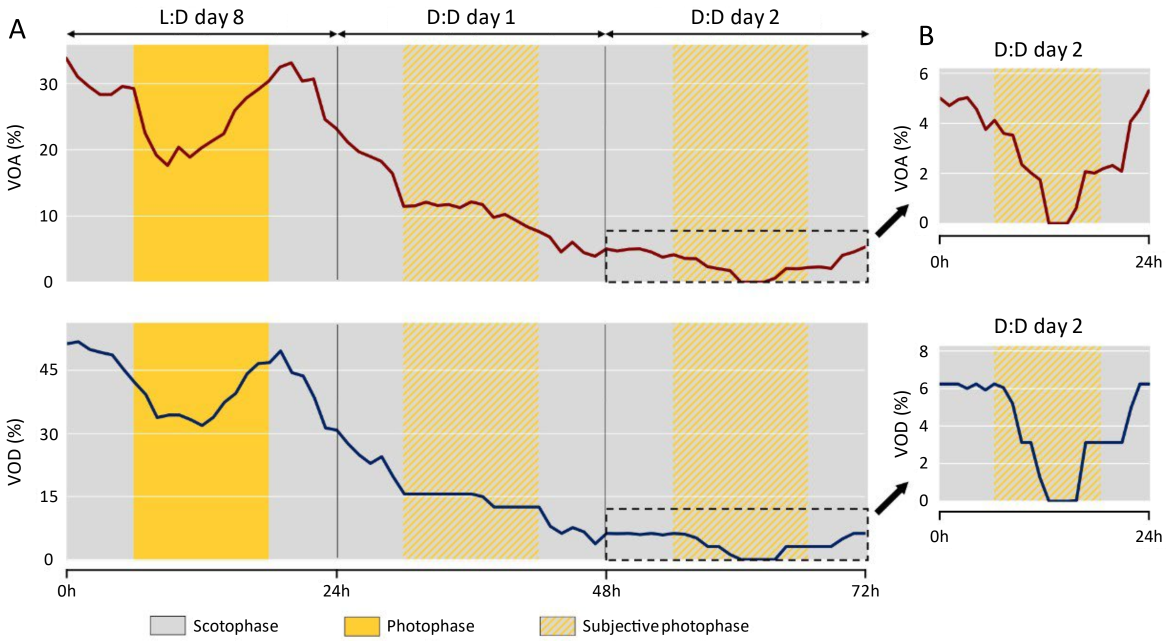

Figure S5. Transition of *Ostrea edulis* valve activity from L:D to D:D. (A) Daily profile of mean VOA (red line) and mean VOD (blue line) during the last day of L:D (Series 1) and the two first days of D:D (Series 2) (B) Zoom on the mean VOA and VOD profiles during the second day of D:D. Grey areas represent scotophase, yellow areas represent photophase, yellow hatched areas represent subjective photophase. n = 16-32.
